# High PD-L2 Predicts Early Recurrence of ER-Positive Breast Cancer

**DOI:** 10.1200/PO.21.00498

**Published:** 2023-01-18

**Authors:** Inna Chervoneva, Amy R. Peck, Yunguang Sun, Misung Yi, Sameer S. Udhane, John F. Langenheim, Melanie A. Girondo, Julie M. Jorns, Lubna N. Chaudhary, Sailaja Kamaraju, Carmen Bergom, Michael J. Flister, Jeffrey A. Hooke, Albert J. Kovatich, Craig D. Shriver, Hai Hu, Juan P. Palazzo, Marluce Bibbo, Terry Hyslop, Marja T. Nevalainen, Richard G. Pestell, Serge Y. Fuchs, Edith P. Mitchell, Hallgeir Rui

**Affiliations:** ^1^ Division of Biostatistics, Thomas Jefferson University, Philadelphia, PA; ^2^ Department of Pathology, Medical College of Wisconsin, Milwaukee, WI; ^3^ Department of Medicine, Medical College of Wisconsin, Milwaukee, WI; ^4^ Department Radiation Oncology, Medical College of Wisconsin, Milwaukee, WI; ^5^ Department of Physiology, Medical College of Wisconsin, Milwaukee, WI; ^6^ John P. Murtha Cancer Center, Uniformed Services University, Bethesda, MD; ^7^ Chan Soon-Shiong Institute of Molecular Medicine at Windber, Windber, PA; ^8^ Department of Pathology, Thomas Jefferson University, Philadelphia, PA; ^9^ Center for Health Equity, Sidney Kimmel Cancer Center, Thomas Jefferson University, Philadelphia, PA; ^10^ Pennsylvania Cancer and Regenerative Medicine Research Center, Baruch S. Blumberg Institute, Doylestown, PA; ^11^ The Wistar Cancer Center, Philadelphia, PA; ^12^ Department of Biomedical Sciences, University of Pennsylvania, Philadelphia, PA; ^13^ Department of Medical Oncology, Thomas Jefferson University, Philadelphia, PA

## Abstract

T-cell–mediated cytotoxicity is suppressed when programmed cell death-1 (PD-1) is bound by PD-1 ligand-1 (PD-L1) or PD-L2. Although PD-1 inhibitors have been approved for triple-negative breast cancer, the lower response rates of 25%-30% in estrogen receptor–positive (ER+) breast cancer will require markers to identify likely responders. The focus of this study was to evaluate whether PD-L2, which has higher affinity than PD-L1 for PD-1, is a predictor of early recurrence in ER+ breast cancer.

**METHODS:**

PD-L2 protein levels in cancer cells and stromal cells of therapy-naive, localized or locoregional ER+ breast cancers were measured retrospectively by quantitative immunofluorescence histocytometry and correlated with progression-free survival (PFS) in the main study cohort (n = 684) and in an independent validation cohort (n = 273). All patients subsequently received standard-of-care adjuvant therapy without immune checkpoint inhibitors.

**RESULTS:**

Univariate analysis of the main cohort revealed that high PD-L2 expression in cancer cells was associated with shorter PFS (hazard ratio [HR], 1.8; 95% CI, 1.3 to 2.6; *P* = .001), which was validated in an independent cohort (HR, 2.3; 95% CI, 1.1 to 4.8; *P* = .026) and remained independently predictive after multivariable adjustment for common clinicopathological variables (HR, 2.0; 95% CI, 1.4 to 2.9; *P* < .001). Subanalysis of the ER+ breast cancer patients treated with adjuvant chemotherapy (n = 197) revealed that high PD-L2 levels in cancer cells associated with short PFS in univariate (HR, 2.5; 95% CI, 1.4 to 4.4; *P* = .003) and multivariable analyses (HR, 3.4; 95% CI, 1.9 to 6.2; *P* < .001).

**CONCLUSION:**

Up to one third of treatment-naive ER+ breast tumors expressed high PD-L2 levels, which independently predicted poor clinical outcome, with evidence of further elevated risk of progression in patients who received adjuvant chemotherapy. Collectively, these data warrant studies to gain a deeper understanding of PD-L2 in the progression of ER+ breast cancer and may provide rationale for immune checkpoint blockade for this patient group.

## INTRODUCTION

Cancer cells frequently upregulate the programmed cell death-1 (PD-1) ligands-1 (PD-L1) or -2 (PD-L2) to inactivate tumor-infiltrating cytotoxic T-cells and evade antitumor immunity.^1^ Targeted immune therapies against the PD-1 axis include monoclonal anti–PD-1 antibodies (eg nivolumab, pembrolizumab, and cemiplimab), which block PD-1 receptor interactions with both PD-L1 and PD-L2, and antibodies that bind PD-L1 (eg atezolizumab) and selectively prevent PD-L1 binding to PD-1.^2,3^ Both approaches have provided antitumor effects in several cancer types,^4‐12^ including frequently durable responses in triple-negative breast cancer (TNBC)^13‐16^ and in 25%-30% of estrogen receptor–positive (ER+) breast cancer.^14,17‐20^ Diverse cancer types including breast cancer can have variable responses to PD-1 axis blockade regardless of their PD-L1 status, including responses seen in PD-L1–negative tumors.^5,8,9,21,22^ For instance, responses to pembrolizumab in the KEYNOTE-086 and ClinicalTrials.gov identifier: NCT03051659 trials in metastatic TNBC were similar in patients with PD-L1–positive and PD-L1–negative tumors (reviewed in Kwa and Adams^23^). The KEYNOTE-522 trial resulted in the recent approval of pembrolizumab in the neoadjuvant chemotherapy setting in TNBC regardless of PD-L1 status.^24^ Collectively, these observations suggest that biomarkers are needed to stratify patients for these therapies, especially for ER+ breast cancer with lower response rates.

**CONTEXT**

**Key Objective**
Programmed cell death-1 (PD-1) inhibitors have been approved for triple-negative breast cancer. Durable responses to PD-1 inhibitors are less common for estrogen receptor–positive (ER+) breast cancers, and markers are needed to identify likely responders. Although most efforts have focused on the immune checkpoint protein PD-L1, the alternative PD-1 ligand, PD-L2, has been largely overlooked. This study used quantitative histocytometry to determine if the immunosuppressive PD-L2 is associated with unfavorable prognosis in ER+ breast cancer.
**Knowledge Generated**
High PD-L2 protein levels in cancer cells was detected in up to one third of therapy-naive ER+ breast tumors and was validated as an independent predictor of early breast cancer recurrence after adjustment for common clinicopathological variables.
**Relevance**
PD-L2 is a therapy-relevant marker and may help identify patients with ER+ breast cancer who are at elevated risk of progression and who may benefit from PD-1 inhibitors.

PD-L2 has 2-6 fold higher affinity for PD-1 than PD-L1.^25^ PD-L2 has been associated with poor prognosis in some solid tumors, including hepatocellular carcinoma and clear cell renal carcinoma.^26^ However, although PD-L2 is frequently expressed by malignant breast epithelia,^27^ the prognostic value of PD-L2 expression in breast cancer remains largely unexplored. To date, two small exploratory studies of PD-L2 as a marker of clinical outcome in TNBC and human epidermal growth factor receptor 2–positive breast cancer yielded largely inconclusive results,^27,28^ and PD-L2 has not been explored in ER+ breast cancer. On the basis of the reported expression of PD-L2 on malignant breast epithelia^27,28^ and its high affinity for PD-1,^25^ we hypothesized that elevated PD-L2 is associated with unfavorable outcome in locoregional ER+ breast cancer. We applied quantitative immunofluorescence histocytometry to correlate levels of PD-L2 on malignant epithelial and stromal cells with clinical outcome in luminal breast cancer in a main patient cohort treated at one medical center (n = 684) and in an independent validation cohort (n = 273) from a separate medical center. One third of ER+ breast tumors expressed high levels of PD-L2 protein in cancer cells, which correlated with shorter progression-free survival (PFS) and remained independently predictive of poor outcome after multivariable adjustment for common clinicopathological variables.

## METHODS

### Study Design and Participants

This retrospective study included a main study cohort of archival formalin-fixed and paraffin-embedded specimens of primary invasive breast cancer from Thomas Jefferson University Hospital, Philadelphia, PA, and an independent validation cohort from the Clinical Breast Care Program (CBCP) at the Walter Reed National Military Medical Center, Bethesda, MD. Specimen and data collections were conducted according to research protocols approved by local Institutional Review Boards. The specimens were unselected consecutive cases with the inclusion criteria of available tumor tissue and clinical outcome data. Patients of any age were eligible for analysis. Patients received postoperative radiotherapy or systemic therapy according to the treating physician's discretion. Tumor specimens were reviewed by central pathologist (J.A.H.), and representative cancer tissue regions were selected for inclusion in tissue microarrays (TMAs; 0.6 mm core diameter; 3DHistech Grandmaster arrayer). Quantitative PD-L2 immunofluorescence-based histocytometry was performed with the operators blinded to patient IDs and clinical outcomes. Some tissue cores were excluded from further analysis if the quality of the staining was deemed substandard. ER positivity was defined according to the definition in use at the time of diagnosis. A small number of patients had duplicated tissue cores. For these patients, the geometric mean of the core-specific PD-L2 expression (median expression in cancer or stroma cells) was used as the marker value. After excluding patients with metastatic disease at diagnosis or with tumors of negative or unknown ER status, the main study cohort included 684 patients diagnosed with ER+ breast cancer between 1988-2005 (median PFS = 114.5 months; range 2-238 months; 120 events [recurrence of invasive disease only]; see Table 1 for clinical characteristics, REMARK diagram in the Data Supplement), and the external validation cohort included 273 patients diagnosed with ER+ breast cancer between 1992-2012 (median PFS = 148 months, range 0.8-297 months; 31 events [recurrence of invasive disease only]; see Data Supplement for clinical characteristics, REMARK diagram in the Data Supplement).

**TABLE 1.**
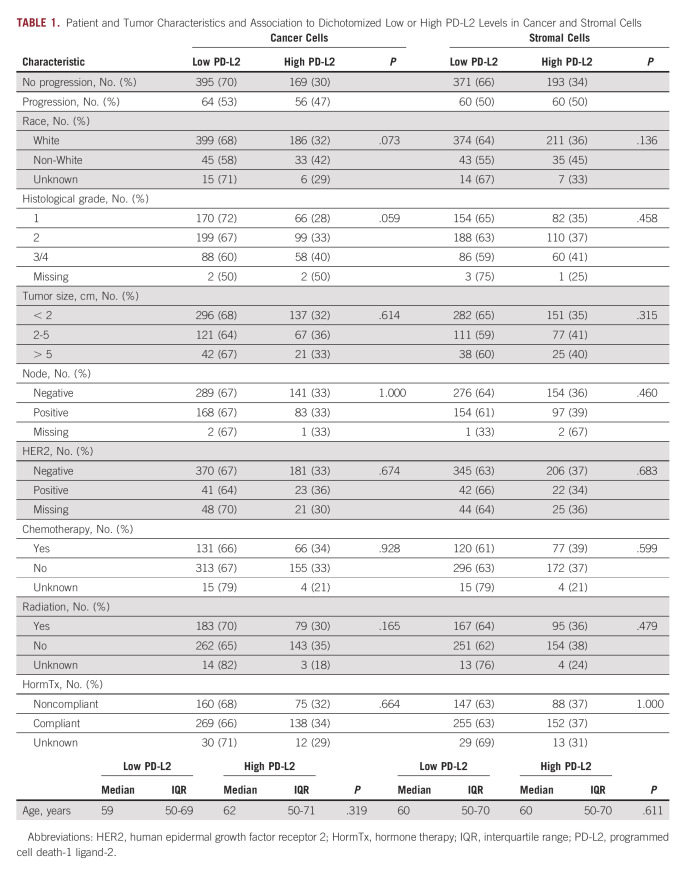
Patient and Tumor Characteristics and Association to Dichotomized Low or High PD-L2 Levels in Cancer and Stromal Cells

### PD-L2 Antibody Validation and Quantitative Immunofluorescence-Based Histocytometry

Immunostaining, slide scanning, and quantitative analysis of digitized images were performed in the laboratory of H.R. in a manner blinded to outcome. Immunostaining for PD-L2 was performed on an autostainer (Dako-Omnis) using rabbit polyclonal antibody (Sigma-Cat#SAB3500395, 1:200) followed by linker and HRP-conjugated secondary antibody (Dako-Cat#K4003) and visualized with Cy5-tyramide as substrate, multiplexed with anti–pan-cytokeratin antibody (Dako-Cat# M3515) with Alexa-555–labeled secondary antibody (Thermo-Fisher-Cat#A21422) to identify cancer cells, finalized by 4′,6-diamidino-2-phenylindole (DAPI) counterstain to visualize cell nuclei as previously described.^1-3^ Stained slides were digitized at 20× magnification on the Scanscope scanner (Leica/Aperio, Deer Park, IL), and fluorescent images were captured in three channels (Cy5-Alexa555-DAPI). Digitized images were analyzed by Tissue Studio (Definiens, Munich, Germany) and cytoplasmic expression signals for PD-L2 immunoreactivity were computed for individual cancer cells identified by pan-cytokeratin–positive mapping and in stromal cells for each tumor core. PD-L2 protein expression was computed as the median cytoplasmic cell signal intensity in the cancer cell and stromal cell populations of each tumor. Immunostaining using the PD-L2 antibody SAB3500395 has been extensively reported to detect PD-L2 in malignant tumors.^4-6^ Our own validation verified (1) specific detection of PD-L2 in FFPE pellets of positive control HEK-293 cells and not in matched PD-L2–negative HEK-293 cells (Data Supplement), (2) PD-L2 expression in placenta-positive control tissue, with staining pattern comparable with that of three distinct PD-L2 antibodies (Abcam-Cat#ab214221, R&D-Cat#MAB1224, and Sigma-Cat#HPA013411; Data Supplement), and (3) reproducible measurement of PD-L2 levels in 40 breast cancer specimens in adjacent sections of a control TMA that were stained on two different days (Data Supplement).

### Statistical Analyses

PFS was defined as the time from diagnosis to the evidence of local, regional, or distant recurrence or death from breast cancer. Patients without progression were censored at the last follow-up time. In the main study cohort, there were 120 patients with disease progression. With respect to hormone treatment, patients were classified as noncompliant if hormone treatment was recommended but patients did not comply. In the main study cohort, median PD-L2 levels in cancer or stromal cells were dichotomized into high versus low (separately for cancer and stromal expression) using the survival tree model with 10-fold cross-validation (R package rpart).^7^ The internal validation of the optimal cutpoints was performed using the bootstrap optimism correction procedure.^29^ All hazard ratios (HRs) reported for the main study cohort are optimism-corrected for dichotomized PD-L2 in the univariate and multivariate Cox models for PFS. Multiple imputations were used since the clinicopathologic covariates information was missing for some patients (0.6%-10% for various clinicopathological and treatment covariates, see Table 1). Forty imputed data sets were created using the multivariate imputation by chained equations algorithm. For each covariate, missing values were imputed by univariate models for corresponding outcome type using the Fully Conditional Specification.^30^ The bootstrap optimism correction algorithm was applied to each imputed data set. Then, results for all imputed data sets were averaged using the Rubin rule.^31^ The detailed steps of the bootstrap optimism correction algorithm are provided in the Data Supplement. The association between PD-L2 (dichotomized using the median of the cutpoints in all bootstrap samples) and categorical clinicopathological factors was evaluated using the Fisher exact test or its extension for more than two categories. For the continuous age variable, the two-sample Wilcoxon signed-rank test was used. The multivariable Cox proportional hazards model included the standard clinicopathological prognostic factors of PFS: age, race (White *v* non-White), histologic grade, node status, tumor size (< 2 cm, 2-5 cm, or > 5 cm), radiation therapy, chemotherapy, and hormone therapy compliance (*v* the reference category including patients who had hormone therapy and patients with hormone therapy not indicated). The first-order interactions were considered. The proportional hazards assumption was evaluated for all covariates in the multivariable Cox model, and covariates that violated the proportional hazard assumptions were incorporated in the models as strata variables. For analysis of the external validation cohort, optimism-corrected cutpoints for cancer and stromal cell PD-L2 levels derived from the main study cohort were applied to dichotomize cancer cell and stromal cell PD-L2 levels for evaluation using the univariate Cox proportional hazards model. The multivariable Cox models were not considered for the external validation cohort because of a limited number of progressions and incomplete treatment information. Data were analyzed in R^8^ and SAS 9.4.

## RESULTS

### PD-L2 Expressed in Cancer Cells of ER+ Breast Tumors Is Associated With Unfavorable Outcome

**FIG 1.**
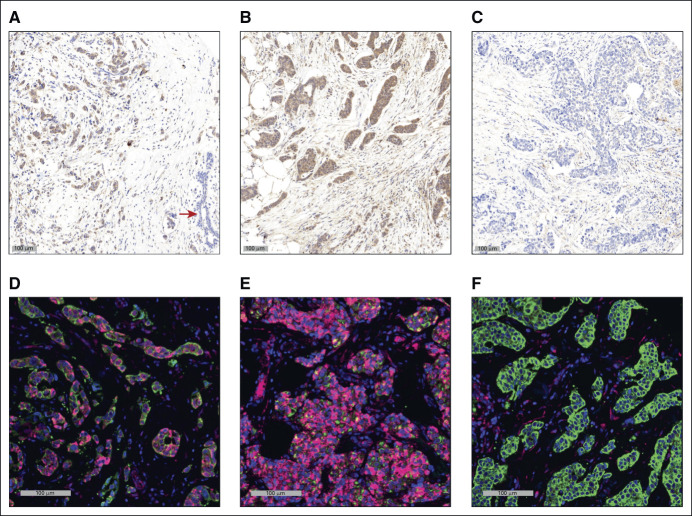
PD-L2 immunostaining in ER+ breast cancer. (A-C; brown signal) Immunohistochemistry of PD-L2 using diaminobenzidine chromogen and (D-F; red signal) immunofluorescence. A, B, D, and E are different examples of ER+ tumors with high cancer cell expression of PD-L2, whereas C and F are examples with low cancer cell PD-L2 expression. Note the absence of PD-L2 in nearby normal duct epithelium (A; arrow). Tumors stained for PD-L2 (D-F; red signal) by the immunofluorescent protocol used for quantification were counterstained for pan-cytokeratin (green signal) to help identify cancer cells and DAPI (blue signal) to identify cell nuclei. Scale bar represents 100 µm. DAPI, 4′,6-diamidino-2-phenylindole; ER+, estrogen receptor–positive; PD-L2, programmed cell death-1 ligand-2.

**FIG 2.**
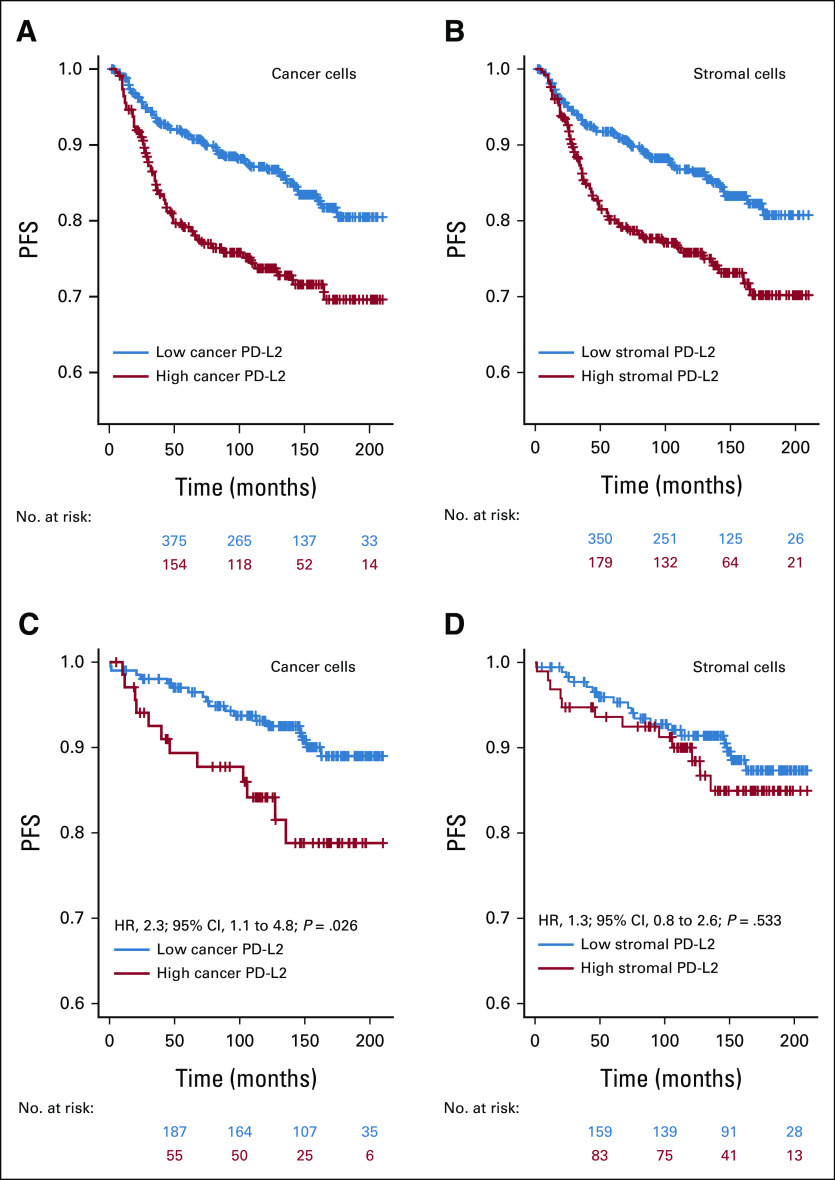
Kaplan-Meier curves for PFS in patients with ER+ breast cancer by dichotomized median PD-L2 expression. The median PD-L2 expression was dichotomized in (A) cancer cells and in (B) stromal cells using the median of the distribution of cutpoints obtained in 500 bootstrap samples from the main analysis cohort. (C) The association between short PFS and high cancer cell PD-L2 levels was validated in the independent external cohort, (D) whereas the association between short PFS and high stromal cell PD-L2 levels did not validate. ER+, estrogen receptor–positive; PD-L2, programmed cell death-1 ligand-2; PFS, progression-free survival.

PD-L2 protein levels in cancer cells and stroma cells were measured retrospectively by fluorescence-based immunohistochemical staining and quantitative image histocytometry in ER+ breast cancer specimens from a total of 684 patients using a validated immunohistochemistry protocol (Figs 1A-1F and Data Supplement). The optimal cutpoints for dichotomization between high and low PD-L2 expression were very similar for the cancer cells (immunofluorescence level = 8.33; 95% CI, 8.05 to 8.44) and stromal cells (immunofluorescence level = 8.16; 95% CI, 7.48 to 8.71). Shorter PFS was associated with high PD-L2 expression in both cancer cells (HR, 1.8; 95% CI, 1.3 to 2.6; *P* = .001; Fig 2A) and stromal cells (HR, 1.5; 95% CI, 1.1 to 2.2; *P* = .018; Fig 2B). Disease recurrence was 25% in cases with high cancer cell PD-L2 levels (56 of 225) and 24% in cases with high stromal cell PD-L2 levels (60 of 253) compared with 14% in cases with low PD-L2 levels (64 of 459 in cancer cells or 60 of 431 in stromal cells). Neither cancer cell nor stromal cell PD-L2 levels were significantly associated with any of the known clinicopathological factors (Table 1). Overall, 33% of ER+ breast tumors were classified as having high PD-L2 levels in the malignant epithelium and 37% of ER+ breast tumors were classified as having high PD-L2 levels in stromal cells. A positive correlation was observed between PD-L2 levels in malignant and stromal cells (*r* = 0.76; 95% CI, 0.72 to 0.79; Data Supplement) and combined high PD-L2 levels in both malignant epithelium and stromal cells were associated with shorter PFS (HR, 1.9; 95% CI, 1.3 to 2.8; *P* = .002; Data Supplement).

The observed association of high PD-L2 levels with short PFS in the main study cohort was then tested in an independent validation cohort of 273 patients. As observed in the main study cohort, PD-L2 levels in both malignant and stromal cell compartments were independent of any reported clinicopathological features (Data Supplement). The optimal cutpoints for dichotomized high and low PD-L2 protein expression that were derived in the main study cohort were applied to the validation cohort, which confirmed the shorter PFS associated with high PD-L2 in the malignant epithelium (HR, 2.3; 95% CI, 1.1 to 4.8; *P* = .026; Fig 2C) but not in the stromal cells (HR, 1.3; 95% CI, 0.8 to 2.6; *P* = .533; Fig 2D).

### PD-L2 Levels in Cancer Cells Is an Independent Predictor of Unfavorable Clinical Outcome in ER+ Breast Tumors

High levels of PD-L2 in cancer cells was further evaluated and found to be an independent prognostic factor in ER+ patients of the main cohort after adjustment for common clinicopathological variables and internal validation using bootstrap-based optimism correction (HR, 2.0; 95% CI, 1.4 to 2.9; *P* < .001; Table 2). As observed in univariate analyses, the stromal cell PD-L2 was a weaker albeit significant predictor of PFS (HR, 1.5; 95% CI, 1.03 to 2.1; *P* = .035; Data Supplement). In addition, as observed in univariate analyses, high PD-L2 levels in both cancer and stromal cells were associated with elevated risk of progression compared with patients with low cancer cell and/or low stroma cell PD-L2 (HR, 1.9; 95% CI, 1.3 to 2.7; *P* = .003; Data Supplement). However, the combined PD-L2 signals did not exceed the risk associated with the cancer cell-specific PD-L2 signal in ER+ breast cancer (HR, 2.0; 95% CI, 1.4 to 2.9; *P* < .001; Table 2). Parallel analysis of PD-L2 levels in ER-negative breast tumors showed higher PD-L2 levels in ER-negative than in ER+ breast tumors in both cancer (*P* < .001) and stromal cells (*P* < .001), but PD-L2 levels of a considerable fraction of ER+ tumors overlapped with ER-negative tumors (Data Supplement). Applying the same methodologies as for ER+ tumors to derive optimal cutpoints for PD-L2 in cancer and stromal cells to ER-negative tumors did not yield any statistically significant associations with PFS (data not shown).

**TABLE 2.**
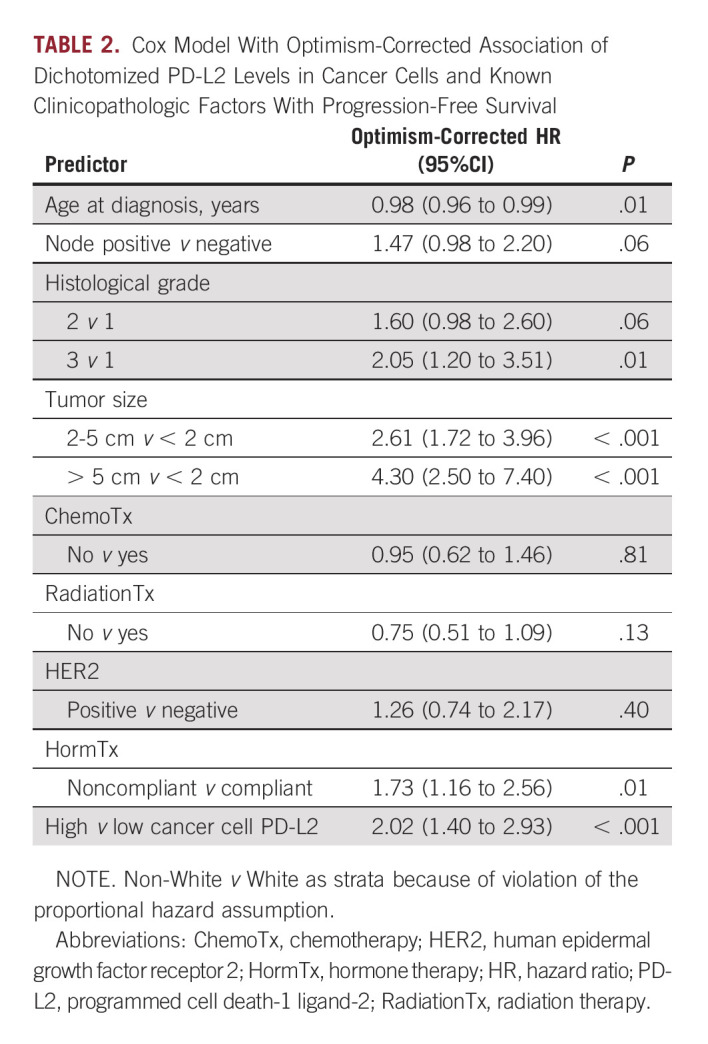
Cox Model With Optimism-Corrected Association of Dichotomized PD-L2 Levels in Cancer Cells and Known Clinicopathologic Factors With Progression-Free Survival

### PD-L2 Is a Strong Predictor of Unfavorable Clinical Outcome in Patients With ER+ Breast Cancer Treated With Adjuvant Chemotherapy

**FIG 3.**
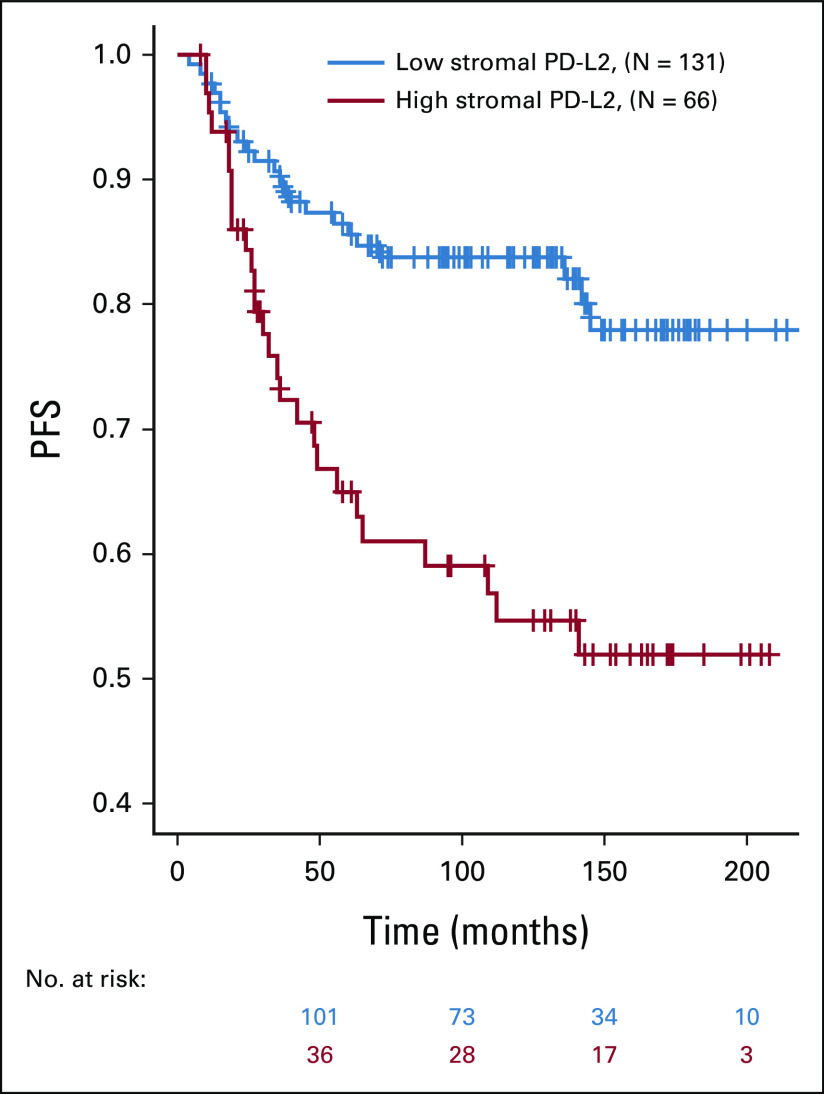
Kaplan-Meier curves for PFS by dichotomized median cancer PD-L2 expression in patients with ER+ breast cancer treated with adjuvant chemotherapy. Univariate estimates of PFS curves. ER+, estrogen receptor–positive; PD-L2, programmed cell death-1 ligand-2; PFS, progression-free survival.

There is a growing consensus that immune checkpoint inhibitors can synergize with chemotherapy in many solid tumors.^32,33^ Although the interactions between PD-L2 markers in ER+ tumors and other clinicopathological covariates were not statistically significant, the interaction between chemotherapy and high cancer cell PD-L2 was potentially important (HR, 1.9; 95% CI, 0.9 to 4.0; *P* = .091). Therefore, a multivariable Cox model that included the interaction between chemotherapy and cancer cell PD-L2 expression was used to evaluate PFS in ER+ breast cancer patients with or without chemotherapy (Data Supplement). This model indicated a larger effect of high PD-L2 in patients who received chemotherapy (HR, 3.0; 95% CI, 1.7 to 5.3; *P* < .001) while the effect of high PD-L2 in patients who did not receive chemotherapy was not statistically significant (HR, 1.43; 95% CI, 0.9 to 2.3; *P* = .156). Separately, we performed subgroup analysis of PD-L2 within the 197 patients with ER+ breast cancer who received adjuvant chemotherapy. In univariate optimism-corrected analysis, high cancer cell PD-L2 was a strong predictor of PFS (HR, 2.5; 95% CI, 1.4 to 2.9; *P* = .003; Fig 3), whereas stromal cell PD-L2 was somewhat weaker predictor of PFS (HR, 1.9; 95% CI, 1.1 to 3.3; *P* = .030). Multivariable adjustment for standard clinicopathological risk factors using a parsimonious model fitted to this subgroup of 197 chemotherapy-treated patients (Table 3) confirmed a strong effect of high cancer cell PD-L2 on PFS in patients treated with adjuvant chemotherapy (HR, 3.4; 95% CI, 1.8 to 6.2; *P* < .001). Finally, to explore whether the association between high cancer cell levels of PD-L2 and PFS was related to hormone treatment, we evaluated PD-L2 separately within subgroups of ER+ patients who did or did not receive hormonal treatment. The resulting Kaplan-Meier curves for PFS by dichotomized median cancer cell PD-L2 in patients according to hormone therapy showed comparable association between high cancer cell PD-L2 and PFS in the two groups (Data Supplement).

**TABLE 3.**
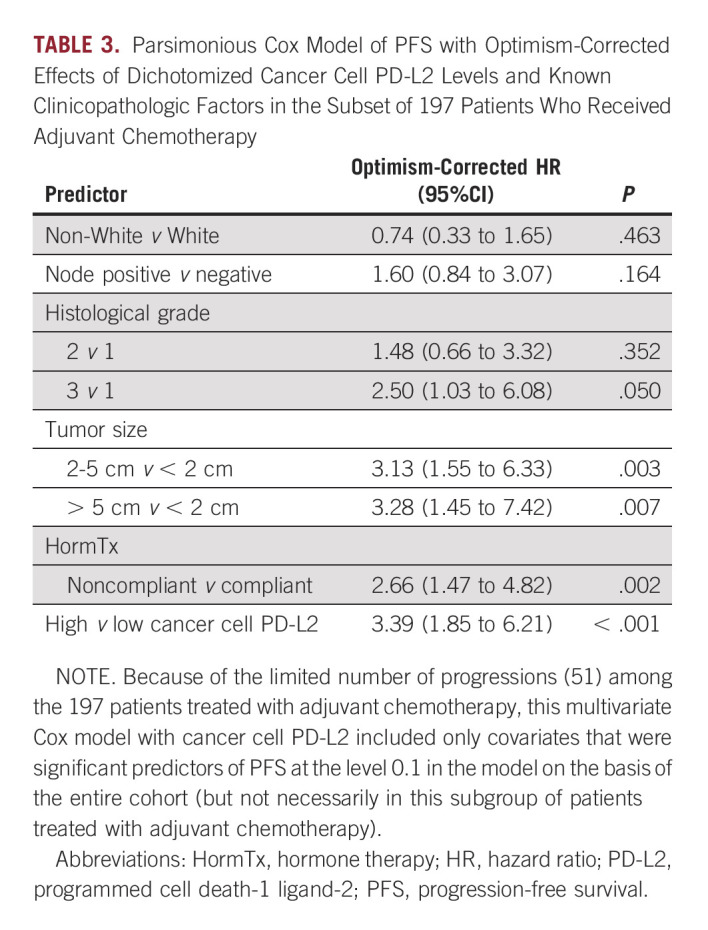
Parsimonious Cox Model of PFS with Optimism-Corrected Effects of Dichotomized Cancer Cell PD-L2 Levels and Known Clinicopathologic Factors in the Subset of 197 Patients Who Received Adjuvant Chemotherapy

## DISCUSSION

In this retrospective study of primary tumors from patients with ER+ breast cancer, including a main study cohort of 684 patients and a validation cohort of 273 patients, we identified high cancer cell levels of PD-L2 protein as an independent predictor of early recurrence after multivariable adjustment for age, race, histological grade, tumor size, node status, and radiation, chemotherapy, and hormone therapy. Among ER+ patients, as many as 33% fell in the high PD-L2 category with an unfavorable outcome. Importantly, this suggests that PD-L2 in cancer cells is an immunosuppressive factor in a substantial fraction of ER+ breast cancers. In support of this notion, efficacy of pembrolizumab was observed in 28% of patients with ER+ breast cancer.^19,20^ Measurement of cancer cell levels of PD-L2 in ER+ breast tumors likely will be informative for response to PD-1 inhibitors or yet to be developed PD-L2 inhibitors. PD-L2 measurement is also relevant for PD-L1–targeted therapy because PD-L1 inhibitors (eg atezolizumab) may not be effective on tumors with predominant PD-L2 expression.

Most if not all clinical trials of PD-1 inhibitors have thus far focused on PD-L1 tumor expression for patient eligibility. However, attempts to correlate responses with PD-L1 have generally failed, although pCR rates were higher in PD-L1–positive patients in the KEYNOTE-522 trial^19,20,24,34^ while PD-L2 has largely been overlooked.^23^ Considering the reported 2-6 fold greater affinity of PD-L2 for PD-1 than PD-L1^25^ and our new data documenting frequent high expression of PD-L2 on ER+ breast cancer cells, PD-L2 expression may be at least as important as PD-L1 in ER+ breast cancer. PD-L2–mediated immune evasion may underlie responses observed in the I-SPY2 trial, where pembrolizumab combined with neoadjuvant chemotherapy was effective in ER+/human epidermal growth factor receptor 2–negative breast cancers as well as in TNBC.^19,20^

Strengths of this study include the large number of patients analyzed with extensive clinical follow-up, the objective and quantitative methodology^35^ used to assess PD-L2 levels, the adjustment for optimistic bias by optimal cutpoint selection, validation of PD-L2 in an independent patient cohort, and multivariable adjustment for common clinicopathological parameters. To our knowledge, this is the most comprehensive and the only quantitative analysis of PD-L2 expression in breast cancer to date. Previous studies of PD-L2 in smaller breast cancer cohorts reported frequent PD-L2 expression consistent with our data but lacked follow-up data or may not have been sufficiently powered.^27,28,36^

Limitations of this study include the retrospective analyses. As patients in the main cohort were diagnosed from 1988 to 2005 and the validation cohort from 1992 to 2012, they received radiation and systemic therapy on the basis of the standard of care at the time of diagnosis, which differs from contemporary therapy because of technical and pharmacological progress and new predictive diagnostics (eg, OncotypeDx, MammaPrint). Second, ER status for patients were classified by pathologists using pre-2010 guidelines of 5% or 10% cutoff for ER positivity instead of the more stringent 1% cutpoint,^37^ thus influencing therapy. However, none of the patients received confounding PD-1 axis inhibitors. Third, TMAs were used for these studies, which provide limited sampling of each tumor. Confirmation of our data in biopsies and whole-tissue sections will be needed. Nonetheless, more extensive tumor sampling is expected to provide more reliable data and would likely strengthen the association between PD-L2 and clinical outcome. Finally, data on both PD-L2 and PD-L1, including their coexpression and distribution across cancer cells and infiltrating immune cell types, are expected to provide complementary value to the cancer cell PD-L2 marker in ER+ breast cancer, despite analytic performance issues of PD-L1 immunohistochemistry assays.^38-43^

Collectively, our study indicates that high levels of PD-L2 identify up to one third of patients with ER+ breast cancer with more aggressive disease and who may benefit from PD-1 inhibitors. PD-L2 expression may contribute to the discordance between PD-L1 levels and tumor response to PD-1 axis inhibitors. This study has motivated activation of a phase II clinical trial of the PD-1 inhibitor cemiplimab for patients with high-risk ER+ or TNBC expressing either PD-L1 or PD-L2 (Chaudhary; ClinicalTrials.gov identifier: NCT04243616). Future focus will be on independent validation and expanded dual analysis of PD-L1 and PD-L2 as predictive markers for PD-1 inhibitors in breast cancer.

## Data Availability

The data sets used and analyzed for the current study are available from the corresponding author on request.
